# CARE Model Assessment for school-age children who stutter: An overview and preliminary findings

**DOI:** 10.3389/fpsyg.2026.1736190

**Published:** 2026-06-05

**Authors:** Courtney T. Byrd, Geoffrey A. Coalson, Edward G. Conture

**Affiliations:** Arthur M. Blank Center for Stuttering Education and Research, The University of Texas at Austin, Speech, Language, and Hearing Sciences, Austin, TX, United States

**Keywords:** children, stuttering-affirming, strengths-based assessment, neurodiversity-affirming, adverse impact, communication, resiliency, community-based participatory research

## Abstract

This article details the community-based participatory research development of the CARE Model Assessment instrument, which measures the Blank Center CARE Model’s four primary components: Communication, Advocacy, Resiliency, and Education (CARE). The CARE Assessment instrument is not designed to diagnose stuttering; rather, it assumes an a priori diagnosis and provides a strengths-based evaluation of the four CARE Model components. It is intended for use in individuals diagnosed with stuttering who may receive stuttering-affirming treatment. The CARE Assessment instrument provides an evaluation framework clinicians can use to inform and construct a strengths-based treatment plan. To develop this instrument, the authors conducted two related studies. Study 1 established and refined the initial Assessment tool, resulting in the Revised CARE Assessment instrument (the current version). Subsequently, Study 2 objectively examined the psychometric properties of this revised instrument using a sample of school-age children (*N* = 107; 8–17 years of age) and their caregivers (*N* = 107). This study examined participants’ responses to establish test-retest reliability, divergent validity, and redundancy across the model’s four components. Together, the findings of Studies 1 and 2 provide empirical evidence that the CARE Assessment instrument yields meaningful qualitative and quantitative insights into communication, advocacy, resilience, and knowledge relative to stuttering from the responses of school-age children, their caregivers, and clinicians.

## 1. Assessment versus diagnosis

**FIGURE 1:**
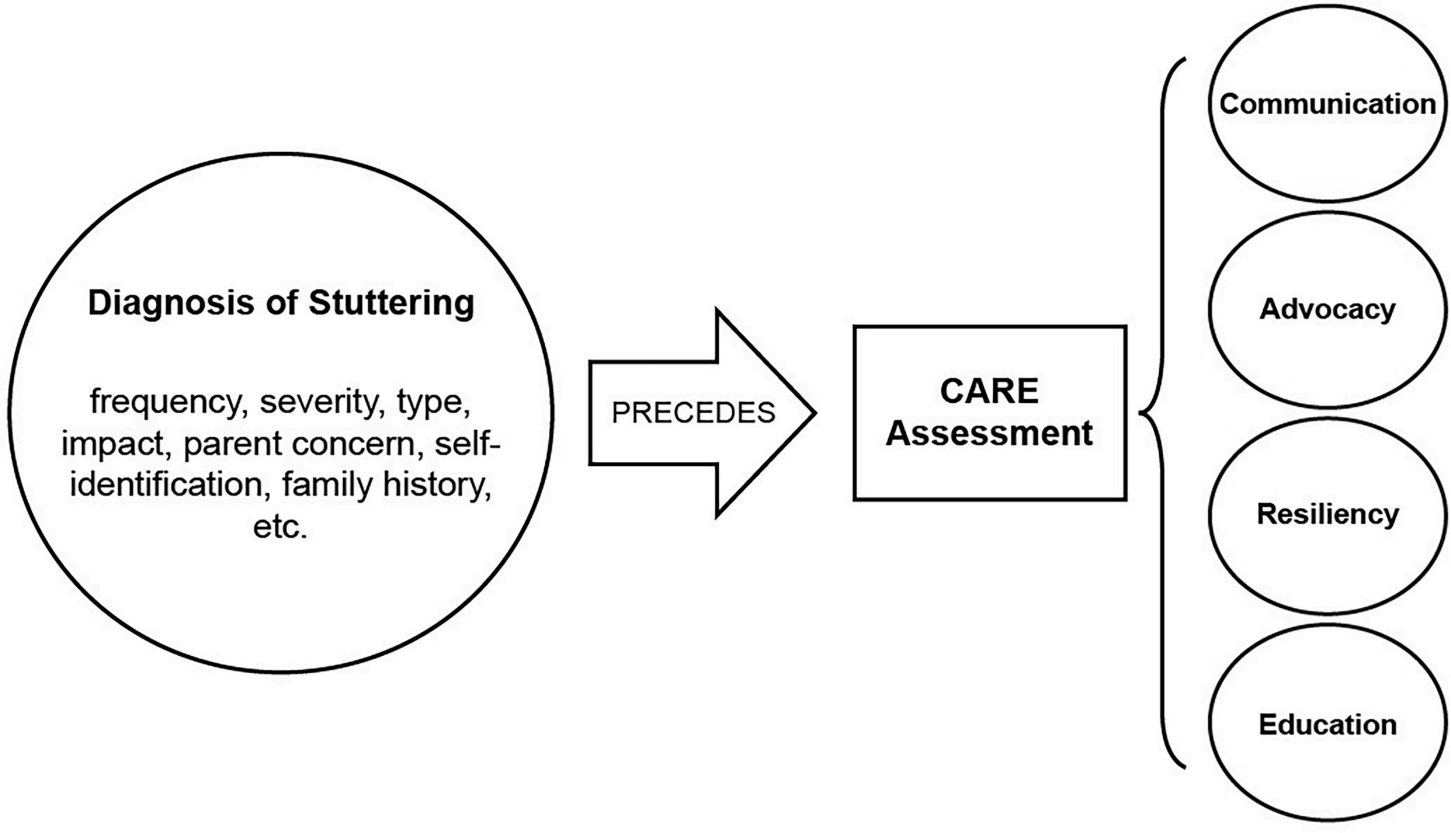
The temporal relationship between (1) the elements typically involved in the diagnosis of stuttering and (2) the CARE Assessment instrument and its four components.

While the constructs of assessment and diagnosis are related and sometimes used synonymously, we suggest that they can be decoupled, particularly in the context of stuttering. The term “diagnosis” is most appropriate for determining whether stuttering is present and for classifying its type, frequency, and severity. Diagnosis typically occurs prior to treatment for stuttering. We are not questioning the necessity of evidence-based stuttering diagnostic procedures. Instead, we are proposing that following a diagnosis of stuttering, a strengths-based treatment plan can be developed using the Blank Center CARE™ Model Assessment instrument (see Figure 1). The present paper covers the community-based participatory research related to the instrument’s development and preliminary findings.

**FIGURE 2:**
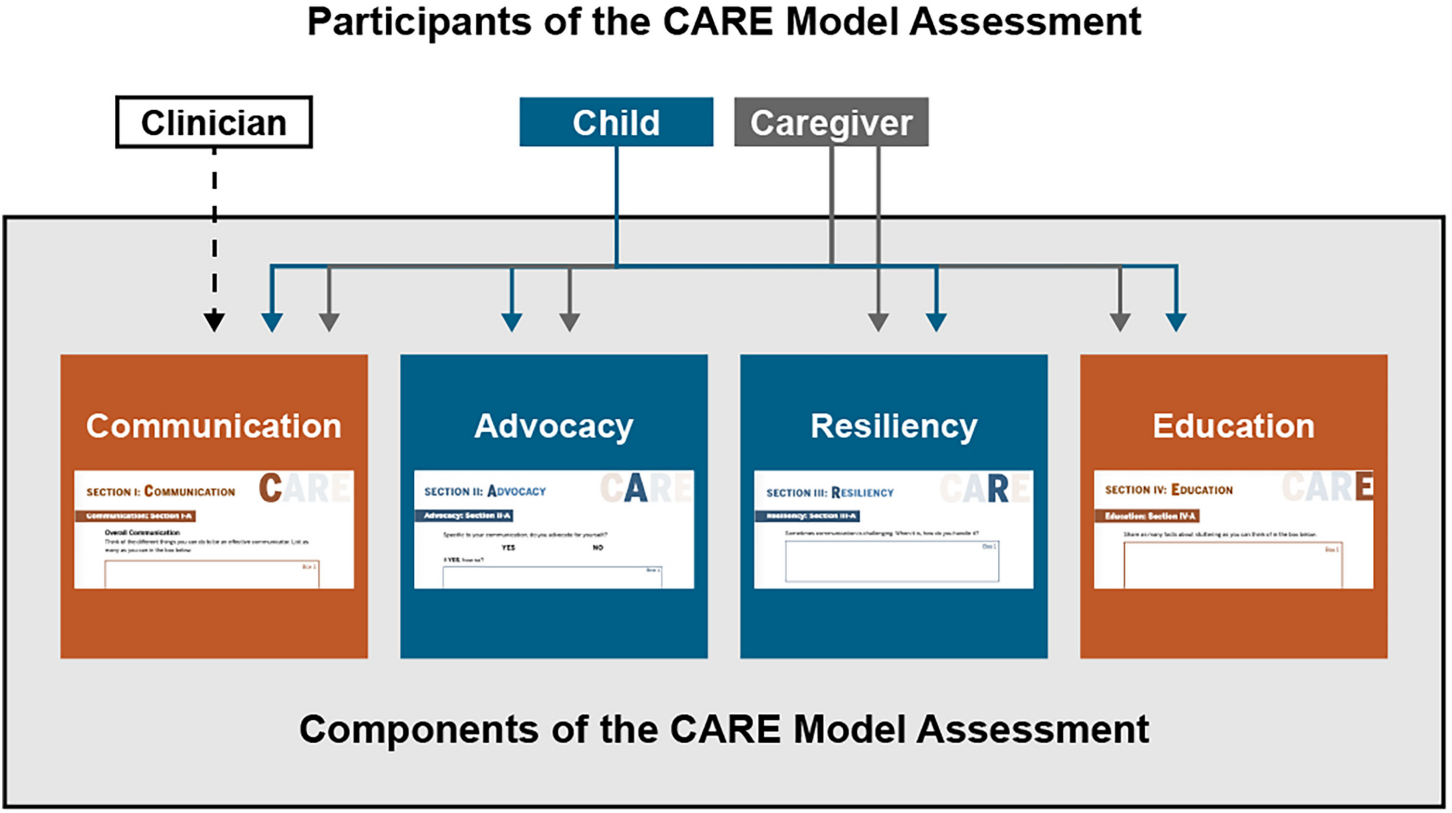
The participants (i.e., child, caregivers, clinicians) and the four components (i.e., Communication, Advocacy, Resiliency, Education) of the Revised (2026) CARE Model Assessment instrument. Clinicians only provided ratings for the Communication component.

The CARE Model Assessment instrument quantitatively and qualitatively measures the four primary components of the Blank Center CARE™ Model (hereafter, the CARE Model): Communication, Advocacy, Resilience, and Education (Byrd et al., 2024a). The CARE Assessment instrument measures these components by collecting response data from individuals who stutter (e.g., school-age children), their caregivers, and their clinicians. It is important to note, however, that this assessment does not represent a norm-based determination of the CARE Model’s four components. Rather, the instrument provides an individualized, evidence-based understanding of a child’s unique strengths in each of the four CARE Model’s components, from the perspectives of (1) the child, (2) the caregiver, and (3) the clinician (see Figure 2).

The results of empirical studies make apparent the fact communication can be judged independently from stuttering (e.g., untrained observers: Byrd et al., 2024b; Coalson and Byrd, 2025; individuals who stutter: Coalson et al., 2024; Erickson and Block, 2013; Werle et al., 2021; Winters and Byrd, 2021), and that quality of life can be improved independent of speech fluency (e.g., Beilby et al., 2012; Byrd et al., 2018; Constantino et al., 2020; Cream et al., 2003; Hugh-Jones and Smith, 1999; Menzies et al., 2019; Winters and Byrd, 2024). To our knowledge, the CARE Model Assessment instrument represents the first data-based, strengths-based instrument for children who stutter that allows for evaluation of communication separate from measures of stuttering of frequency and severity. While prevailing measures focus primarily on stuttering behaviors and its potential negative psychosocial impact, the CARE Model Assessment primarily focuses on existing and emerging strengths. Thus, the CARE Model Assessment fills a critical gap in the prevailing stuttering assessments.

To provide context for the CARE Model Assessment (Byrd, 2023a,b) instrument, we briefly describe the theoretical framework on which it is based, the CARE Model (Byrd, 2022, 2023c), below. The current paper focuses solely on its pre-treatment application and does not consider its use during treatment or as a post-treatment outcome measure.

## 2 Brief description of the CARE Model

### 2.1 Communication

The first of the four CARE Model components—Communication—is defined as the process of exchanging, imparting, or providing information, understanding, and support (Byrd, 2023c; Byrd et al., 2024a). Within this framework, communication is the keystone of human connection. The CARE Model is based, in part, on the belief that perceived communication effectiveness, as evaluated by both the self and others, strengthens human connection and contributes to quality of life (QOL). Numerous behaviors other than fluency contribute to communication effectiveness (e.g., facial expression, speaking rate, organization of spoken content, eye contact, listening, turn-taking, gestures) which are targeted within the CARE Model (for clinical outcomes examining these communication behaviors, see Byrd et al., 2021, 2022, 2025; Coalson et al., 2024). For more information, see Byrd et al. (2024a).

### 2.2 Advocacy

The second of the four CARE Model components—Advocacy—involves the process of apparent, demonstrable, and/or public support or recommendation of a particular idea or point of view (Byrd, 2023c; Byrd et al., 2024a). Within this model, advocacy serves as a mechanism for fostering empathy. In other words, the CARE Model is based, in part, on the belief that self-advocacy helps educate others about stuttering within their environment, thereby mitigating negative stereotypes and empowering the self (e.g., Byrd et al., 2017; Coalson et al., 2026; Young et al., 2022, 2023). For additional discussion, see Byrd et al. (2024a).

### 2.3 Resiliency

The third of the four CARE Model components—Resilience—involves the process of recovering, adapting or returning to baseline following adversity (Byrd, 2023c; Byrd et al., 2024a). Within the CARE Model, resilience is considered germane to an individual’s mental wellbeing (e.g., Croft and Byrd, 2020, 2023; Werle et al., 2025). As such, this framework posits that resilience functions as a buffer between (a) the external negative reactions a speaker may encounter due to stuttering (e.g., Werle et al., 2025) and (b) the speaker’s subjective (i.e., self) appraisal of their QOL (e.g., Byrd et al., 2016; Young et al., 2025). For further insight, see Byrd et al. (2024a).

### 2.4 Education

The fourth of the four CARE Model components—Education—is defined as an “enlightening experience that facilitates learning and future teaching” (Byrd, 2023c; Byrd et al., 2024a, p. 6). The model frames education as fundamental to an individual’s growth and holds that it involves increasing an individual’s knowledge of stuttering, communication, and misconceptions related to both. This strengthened understanding is thought to help deconstruct or prevent the development of internalized stereotypes in those who stutter. Research indicates that children (Vanryckeghem et al., 2005) report experiencing such internalized stereotypes early in life, as do adults (Boyle et al., 2023), and their caregivers (Winters and Byrd, 2024). For a detailed review, see Byrd et al. (2024a).

## 3 Study 1—developing the Revised (2026) version of the CARE Model Assessment instrument based on the Initial (2023) version

### 3.1 Purpose

Study 1 aimed to provide an evidence-based, individualized understanding of a child’s existing and emerging strengths across the four components of the CARE Model (i.e., Communication, Advocacy, Resiliency, and Education). Each child’s scores for these components are intended to assist clinicians in establishing individual, strengths-based treatment goals for progress measurement and monitoring.

### 3.2 General format of the Initial (2023) CARE Model Assessment instrument

The initial version of the CARE Assessment instrument, which included a strengths-based determination of the four components of the CARE Model, was substantially longer than the revised (current) form. Consistent with the current format, the initial CARE Model Assessment comprised four sections (I, II, III, and IV) that assessed knowledge and skills for each of the four CARE Model components. However, the original instrument included 221 questions, whereas the revised version comprises 90 questions. Specific details regarding the four sections in the initial format are presented below.

### 3.3 Specific format of the Initial (2023) CARE Model Assessment instrument’s four sections—communication, advocacy, resiliency, education

The Communication section (I) comprised a 99-item questionnaire assessing the individual’s communication knowledge and skills (0–10-point Likert scale: 0 = lesser strength, 10 = greater strength).

The Advocacy section (II) comprised a 28-item questionnaire evaluating the individual’s advocacy knowledge and skills (0–10-point Likert scale: 0 = lesser strength, 10 = greater strength).

The Resiliency section (III) comprised a 48-item questionnaire on the individual’s resilience (0–10-point Likert scale: 0 = lesser strength, 10 = greater strength).

The Education section (IV) consisted of a 46-item questionnaire about the individual’s education on or knowledge of stuttering (0–10-point Likert-scale, 0 = lesser strength, 10 = greater strength).

The data collection protocol was approved by The University of Texas at Austin Institutional Review Board (IRB: 2015-44-0055). All data collected during the development phase, as well as for the initial and revised versions of the CARE Model Assessment, were obtained after participants provided informed consent.

### 3.4 Participants and methods

Participant feedback on the initial version of the CARE Model Assessment instrument was collected from four groups, including (1) school-age children, *N* = 58 (8–17 years of age); (2) speech-language pathologists (SLPs) familiar with the CARE Model, *N* = 7; (3) SLPs unfamiliar with the CARE Model, *N* = 23; and (4) student clinicians, *N* = 32.

As detailed below, we administered the initial CARE Model Assessment instrument multiple times to elicit user feedback and conducted numerous discussions and interviews with these users. We used this feedback to inform the development of the revised (current) version of the CARE Model Assessment instrument. We then evaluated the revised version in Study 2.

### 3.5 Results of Study 1

User feedback provided a variety of suggestions for improvement. Specifically, users indicated that the instrument needed: (1) clearer, more accessible terminology, (2) definitions for key terms, (3) less redundancy, (4) a reduction in overall length, (5) greater insight into self-ratings of situational communication competence, (6) the inclusion of open-ended responses to anchor questionnaire responses, and (7) a transition from the Likert scale to a 0–100 visual analog scale (VAS).

The following paragraph describes the developmental sequence used to improve and refine the Initial (2023) CARE Model Assessment instrument. Based on the feedback from children, teens, and adults who stutter, as well as clinicians, caregivers, and teachers, we identified five areas for modification.

(1) Feedback indicated that the initial version of the CARE Assessment instrument required modification to ensure that the *language and phrasing* within individual items included vocabulary and structure that were more easily understood for school-age populations. We altered items necessary in response to this critique.
(2) Feedback suggested that the instrument needed to include *definitions of terms as well as a reduction in redundancy and length*. It was also suggested that terms central to the Assessment instrument be defined to ensure participants understand the concepts before responding. Additionally, feedback indicated redundancy across individual test questions and a need to reduce administrator and participant effort and time. As mentioned above, the Initial (2023) CARE Model Assessment of the CARE Assessment comprised 221 questions across the four components of the CARE Model and required, on average, 90 min to administer. Based on clinician feedback, we also integrated the scoring directly into the child and caregiver test forms, and developed a standalone administration manual, to improve ease of administration. Participants also noted that in the initial version of the instrument, the Communication section asked about specific competencies across repetitive situations. Thus, for the Revised (2026) CARE Model Assessment instrument, we condensed all components to eliminate such redundancies.
(3) Feedback suggested that a *broader context for assessing communication* was needed. Therefore, we included two structured communication contexts in the revised version: (1) an impromptu oral presentation and (2) an impromptu dyadic interaction. In the revised version, both the child and the clinician rate the child’s overall communication in both contexts, as well as use of specific communication competencies, on a 0–100-point VAS (0 = not at all effective, 100 = extremely effective).
(4) Feedback suggested *broadening the nature of the sampling*. For example, the Communication section of the Initial (2023) CARE Model Assessment collected ratings of communication competence without asking the child or caregiver how they defined it. In the revised version, before the communication questions, both the child and their caregiver are asked to share what they think is required to be a great communicator. Additionally, to further broaden the scope of the sampling, a teacher proxy form was also developed to assess communication and advocacy skills in the classroom.
(5) The feedback also suggested *refining the scale for ratings of communication competence*. Specifically, following the question about what children and caregivers think is required to be a great communicator and their strengths when communicating, the revised instrument now asks them to rate their overall communication competence on a 100-point VAS (0 = not at all effective, 100 = extremely effective). SLPs suggested changing the rating scale from a Likert-based scale to a VAS, and other participants confirmed that it enabled them to be more specific in their ratings.

### 3.6 Brief discussion of Study 1

Taken together, user feedback indicated that the Initial (2023) CARE Model Assessment instrument needed modification regarding:

(1) Language and phrasing
(2) Definition of terms, reduction in redundancy, reduction in length
(3) Context for assessing communication (and the other three components)
(4) The nature of sampling
(5) Scales for rating communication

After we made all the suggested changes to the initial version of the CARE Model Assessment instrument, a revised version was administered by SLPs (familiar and unfamiliar with the CARE Model). We also administered the revised version to a subsample of individuals who had completed the initial version and a subsample of those who had not, soliciting their feedback.

Based on the results of this revised Assessment instrument, we made a few minor wording changes. We also included culturally responsive instructions for the examiner. These instructions were intended to assist examiners in appropriately adapting the questionnaire items to the specific needs of particular examinees (e.g., not evaluating eye contact for those for whom it may not be neurodevelopmentally or culturally applicable). This draft was then reviewed by all stakeholders (i.e., teachers, clinicians, caregivers, children, teens, and adults who stutter) and re-administered by the same familiar and unfamiliar SLP cohorts to the same subsample of participants for additional feedback.

No further substantive revisions were recommended, and the Revised (2026) CARE Model Assessment instrument was examined in Study 2, described below.

## 4 Study 2—inferential statistical analyses of hypotheses regarding the Revised (2026) CARE Model Assessment instrument

### 4.1 Purpose

Study 2 aimed to provide preliminary results from administering the Revised (2026) CARE Model Assessment instrument to groups of (1) school-age children, (2) caregivers, and (3) clinicians, reporting descriptive and inferential statistical findings across groups. The study addressed four testable hypotheses regarding the responses to the (current) revised instrument. Specifically, we tested:

*H1*: Child responses will demonstrate significant test-retest reliability

*H2*: Child and clinician ratings of communication will exhibit high test-retest reliability

*H3*: Communication and stuttering scores will show no significant relationship, supporting divergent validity

*H4*: The four assessment components will show distinct contributions and low redundancy, as evidenced by low multicollinearity

We reasoned that the empirical support—or refutation—of these hypotheses should help clarify both the specific and general implications of the Assessment instrument. The clinicians in Study 2 were certified, licensed SLPs with training in the CARE Model and administration of the Revised (2026) CARE Model Assessment. It is important to reiterate that the following findings relate to the administration of the Revised (2026) CARE Model Assessment instrument independent of any related treatment. Specifically, participants did not have any prior participation history and were not simultaneously participating in CARE Model Treatment. In short, the present paper focuses exclusively on assessment, not treatment.

### 4.2 Method

The following sections outline the format and content of all four components—Communication, Advocacy, Resilience, and Education—of the Revised (2026) CARE Model Assessment instrument. The sequence presented below mirrors the precise order in which these components were presented during administration of the Revised (2026) CARE Model Assessment instrument. The details of each component of the revised instrument are intended to provide the necessary context for interpreting the empirical results of Study 2, including the testing of the four hypotheses mentioned above, which will be described at the end of this section.

#### 4.2.1 Communication

The Revised (2026) CARE Model Assessment instrument’s Communication component section (I) comprises three subsections (i.e., Communication I-A, Communication I-B, and Communication I-C).

The first subsection [Communication (I-A), Free Response & Overall Communication] begins with open-ended questions followed by an overall communication rating. The free response questions capture the participant’s independent description of (1) the factors they perceive as contributing to communication competence, and (2) their current strengths as a communicator. Each child’s subsequent overall communication rating is based on communication competence, self-rated on a 100-point VAS (i.e., 0 = “*I do not communicate very well at all*,” 100 = “*I think I communicate the absolute best”*).

The second subsection [Communication (I-B), Communication Activities] comprises two impromptu communication activities: a dyadic conversation and an oral presentation. The child rates their own overall performance in both communication contexts on a 0–100-point VAS of communication competence (i.e., 0 = I think I didn’t communicate very well at all, 100 = I think I communicated the absolute best). Clinicians also provide ratings of overall communication competence and specific communication competencies on a 0–100-point VAS for each speaking context.

The third subsection [Communication (I-C), Communication Assessment] comprises a 22-item questionnaire about the individual’s communication (items rated on a 0- to 10-point Likert scale: 0 = never to 10 = always; for example, “I never use gestures when communicating” to “I always use gestures when communicating”). This section determines each child’s communication strengths based on two measures developed by the National Communication Association. The first measure of the Communication composite scale involves public speaking conditions (i.e., the Competent Speaker Speech Evaluation Form, with construct validity ranging from 9.19 to 10.67 logits; Morreale et al., 2007). The second measure involves dyadic interactions [i.e., the Conversational Skills Rating Scale; inter-rater reliability (average *r* = 0.79), test–posttest reliability (*r* = 0.78; Spitzberg, 2007)].

The third subsection (I-C) of the composite measure of communication strength captures the core competencies for communication across a variety of speaking contexts (e.g., home, school) and listeners (e.g., friends, caregivers, teachers, siblings). Competencies encompass the elements of communication derived from the NCA and adapted within the CARE Model framework for children who stutter: language (vocabulary, formality, organization), voice (rate, volume, emphasis), body (positioning, movement, gestures), face (eye contact, affect), connection (turn-taking, listener awareness), and stuttering openly (rated on a 0–10 point Likert scale, from 0 = never to 10 = always; e.g., “*I never use my voice to emphasize the most important parts of my message.*” to *“I always use my voice to emphasize the most important parts of my message”*).

##### 4.2.1.1 Construct validity

We employed the Self-Perceived Communication Competence scale (SPCC) (McCroskey and McCroskey, 1988; Richmond et al., 1989) to determine the construct validity of the Communication section of the Revised (2026) CARE Model Assessment instrument. The SPCC assesses an individual’s comfort speaking in four different contexts, (1) *Public* (i.e., giving an oral presentation), (2*) Meeting* (i.e., talking during a large group meeting), (3) *Group* (i.e., talking during a small group meeting), and (4) *Dyad* (i.e., talking one-on-one), and with three different audiences (i.e., strangers, acquaintances, friends). We calculated Pearson’s correlation coefficients between the communication-related Revised (2026) CARE Model Assessment instrument subscales and the SPCC, which influenced the development of the instrument and measures similar constructs.

As expected, findings indicated that the overall communication ratings on the Assessment instrument were significantly correlated with the SPCC (*n* = 68, *r* = 0.51, *p* < 0.001). The Revised (2026) CARE Model Assessment instrument’s self-rated communication during dyadic interaction also significantly positively correlated with the SPCC Dyad subscale (*n* = 67, *r* = 0.28, *p* = 0.021). Likewise, the instrument’s oral presentation was significantly, positively correlated with the SPCC’s public presentation subscale (*n* = 67, *r* = 0.35, *p* = 0.004), meeting subscale (i.e., talk during a large meeting, *n* = 67, *r* = 0.33, *p* = 0.006), and group subscale (*n* = 67, *r* = 0.28, *p* = 0.021). Finally, the Communication subscale of the Revised (2026) CARE Model Assessment instrument showed a significant positive correlation with the SPCC (*n* = 68, *r* = 0.59, *p* < 0.001).

#### 4.2.2 Advocacy

The Revised (2026) CARE Model Assessment instrument’s Advocacy component section (II) comprises two subsections (i.e., Advocacy II-A and Advocacy II-B).

The first subsection [Advocacy (II-A), Free Response Advocacy] includes open-ended as well as closed-ended questions about the various ways in which they advocate for themselves specific to their communication.

The second subsection [Advocacy (II-B), Advocacy Assessment] comprises a 22-item questionnaire about the individual’s current advocacy knowledge and skills and preparedness for use (rated on a 0–10 point Likert scale, from 0 = never to 10 = always; e.g., “*I never tell other people that I stutter in situations that are meaningful to me*” to *“I always tell other people that I stutter in situations that are meaningful to me”*).

#### 4.2.3 Resiliency

The Revised (2026) CARE Model Assessment’s Resilience component, Section (III), comprises two subsections (i.e., Resilience III-A and Resilience III-B).

The first subsection [Resilience (III-A), Free Response Resiliency] includes open-ended questions with regards to how they handle communication challenges as well the degree to which they engage in self-compassion.

The second subsection (Resilience [III-B], Resiliency Assessment) consists of a 23-item questionnaire regarding the individual’s current resilience (rated on a 0–10 point Likert-scale, from 0 = never to 10 = always; e.g., “*I never seek out communication challenges in situations that are meaningful to me*” to “*I always seek out communication challenges in situations most meaningful to me*”).

#### 4.2.4 Education

The Revised (2026) CARE Model Assessment’s Education component, Section (IV), comprises two subsections (i.e., Education IV-A and Education IV-B).

The first subsection [Education (IV-A), Free Response Education] includes open-ended questions on (1) facts about stuttering, (2) common misconceptions about stuttering, and (3) what they wish people knew about stuttering.

The second subsection [Education (IV-B), Education Assessment] comprises a 23-item questionnaire about the individual’s current knowledge of stuttering (True/False/I Don’t Know; accurate responses assigned a score of 10; inaccurate or “I Don’t Know” responses assigned a score of 0).

### 4.3 Participants

#### 4.3.1 Age and gender

As shown in Table 1, the complete Revised (2026) CARE Model Assessment instrument was administered to 107 school-age children who stutter (74 males, 33 females), with a mean age of 11.79 years (*SD =* 2.94 years; range: 8–17 years). Participants were included if they met the following criteria: 8–17 years of age, proficiency in English, prior diagnosis of stuttering, and completion of the CARE Assessment by both the participant and their caregiver.

**TABLE 1:** Demographics of Revised (2026) CARE Model Assessment instrument school-age children (*N =* 107) participants.

Demographic	Male	Female	Total
*N*	74	33	107
Age (y)	11.72 (2.93)	11.94 (2.99)	11.79 (2.94)
Age of onset (y)	5.14 (2.59)	4.91 (2.70)	5.08 (2.62)
Stuttering severity—dyad	3.90 (2.09)	3.42 (2.00)	3.75 (2.06)
Stuttering severity—presentation	3.81 (2.21)	3.51 (2.20)	3.72 (2.20)
Reduced lunch or Medicaid eligible	13	6	19
Race			
American Indian or Alaskan Native	0	0	0
Asian	5	2	7
Black or African American	19	10	29
Native Hawaiian or Pacific Islander	0	0	0
White	49	20	69
Did not report	1	1	2
Ethnicity			
Not Hispanic or Latino	64	25	89
Hispanic or Latino	10	7	17
Did not report	0	1	1
Multilingual	10	6	16

#### 4.3.2 Age of onset and stuttering severity

Table 1 also shows that the participants’ mean age at the onset of stuttering was 5.08 years (*SD =* 2.62). Their stuttering severity rating, assessed using O’Brian et al.’s (2004) 9-point stuttering severity scale (1 = no stuttering, 9 = extremely severe stuttering), was *M =* 3.75 (*SD =* 2.06) for one-on-one, dyadic interactions, and *M =* 3.72 (*SD =* 2.20) for oral presentations.

It should be reiterated that neither the Revised (2026) CARE Model Assessment instrument nor the CARE Model Treatment itself measures, rates, or targets speech fluency (see Byrd et al., 2024a for review). Rather, these data are included solely for cohort description.

#### 4.3.3 Demographics

To test the hypotheses listed in Study 2, we administered the Revised (2026) CARE Model Assessment instrument to school-age children and their caregivers. Table 1 depicts the demographics of the school-age cohort.

##### 4.3.3.1 Race

Of the 107 children, 69 were White (49 males, 20 females); 29 were Black or African American (19 males, 10 females); and seven were Asian. Two participants did not identify their race. None of the children identified as American Indian, Alaskan Native, Native Hawaiian, or Pacific Islander.

##### 4.3.3.2 Ethnicity

Of the 107 children, 89 were not Hispanic or Latino (64 male, 25 female), 17 were Hispanic or Latino (10 males, 7 females), and one participant did not self-identify.^1^

##### 4.3.3.3 Multilingualism

Sixteen of the 107 children were bilingual or multilingual (9 males, 5 females).

#### 4.3.4 Study 2’s four hypotheses

##### 4.3.4.1 Consistency of child responses [i.e., test-retest reliability; Hypothesis 1 (H1)]

Our first hypothesis predicted that scores on all four components—Communication, Advocacy, Resiliency, and Education—of the Revised (2026) CARE Model Assessment instrument would demonstrate a significant statistical correlation when administered to randomly selected samples of school-age children who stutter at two distinct points in time, provided there was no intervening treatment between administrations.

##### 4.3.4.2 Consistency of clinician ratings of communication competence [i.e., test-retest reliability; Hypothesis 2 (H2)]

Our second hypothesis predicted a statistical correlation between the perceptual ratings provided by school-age children who stutter, as well as perceptual ratings of their clinicians, regarding their overall communication competence and their communication competence in two speaking contexts (dyad, presentation) across two administrations of the Revised (2026) CARE Model Assessment.

##### 4.3.4.3 Relation between communication and stuttering [i.e., divergent validity; Hypothesis 3 (H3)]

Our third hypothesis predicted that there would be no statistical association between the clinician-rated communication strength of school-age children who stutter and their clinician-rated stuttering severity.

##### 4.3.4.4 Relation among the four Revised (2026) CARE Model Assessment components [i.e., redundancy using multicollinearity analysis; Hypothesis 4 (H4)]

Our fourth hypothesis predicted that for both children who stutter and their caregivers, there would be statistically significant correlations between the four components (Communication, Advocacy, Resiliency, and Education). However, we hypothesized that these components would not be functionally dependent. Findings in support of H4 would suggest that these four components are statistically interrelated but distinct in their functional influence. That is, while these components may move together, no single component serves as a proxy for another.

We posited that empirical support—or refutation—of these four hypotheses should further our understanding of both the specific and general implications of the Revised CARE Assessment instrument.

### 4.4 Results of Study 2

#### 4.4.1 Preliminary descriptive findings

**FIGURE 3:**
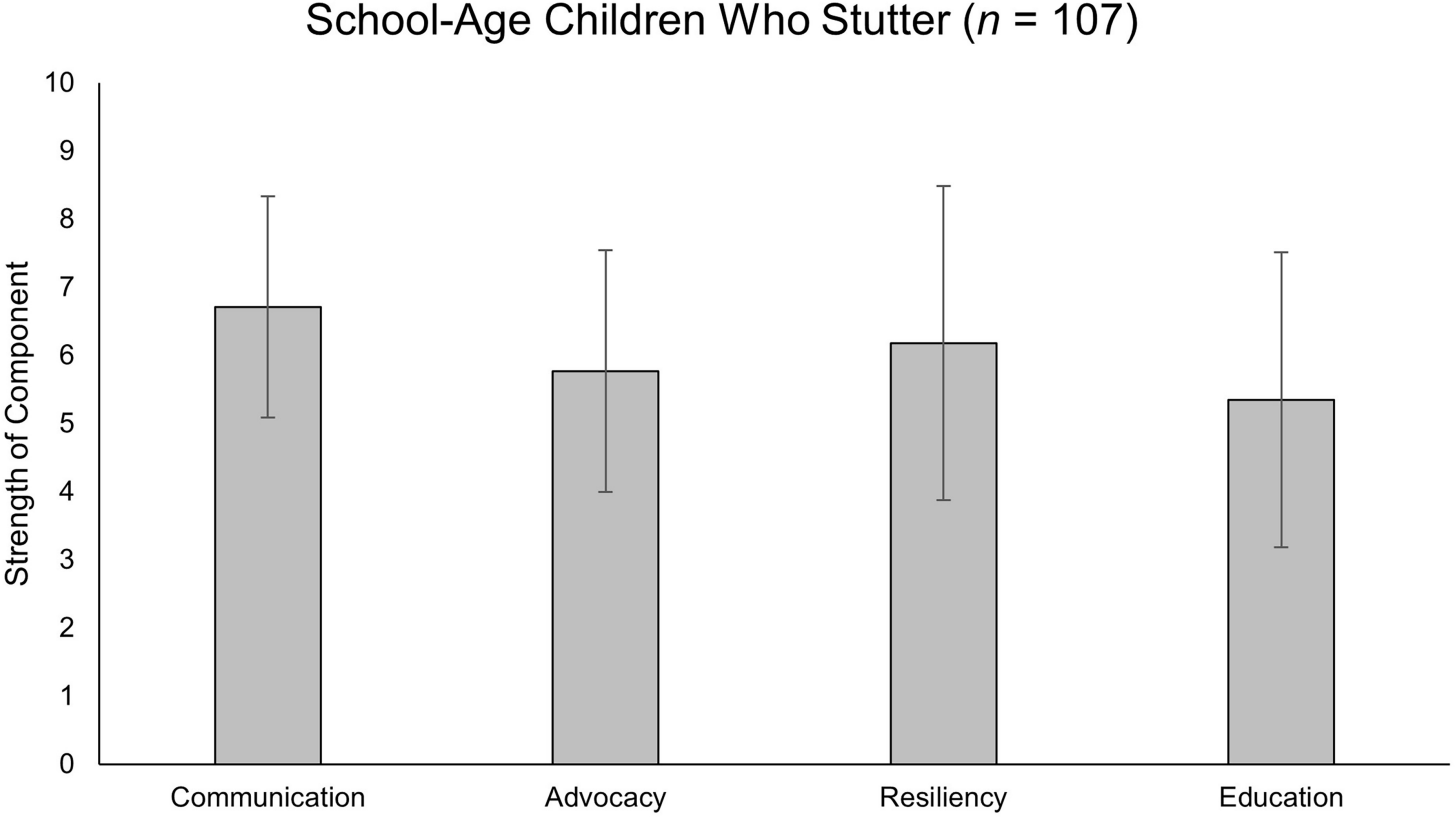
Means and +/- 1 standard deviations for the responses of school-age children who stutter (*N =* 107) to questions relating to each of the four components of the Revised (2026) CARE Model Assessment instrument (0 = low strength and 10 = high strength).

Figure 3 presents the means and standard deviations of the school-age children’s (*N* = 107) responses on all four CARE components [i.e., Communication (I-C), Advocacy (II-B), Resiliency (III-B), and Education (IV-B)] of the Revised (2026) CARE Model Assessment.

An informal evaluation of these responses suggests a high degree of consistency across school-age children’s responses (*M* = 5.3–6.7) for the four components of the CARE Assessment instrument. Self-ratings of overall communication competence [I-A] were *M* = 67.95, *SD* = 20.59 (100-point VAS). Self-ratings of communication competence were *M* = 74.56, *SD* = 20.11 during dyadic interactions (I-B) and *M* = 69.52, *SD* = 24.59 during oral presentations (I-B). Clinician ratings were *M* = 61.31, *SD* = 13.77 for dyadic interactions (I-B) and *M* = 59.79, *SD* = 16.03 for oral presentations (I-B).

**FIGURE 4:**
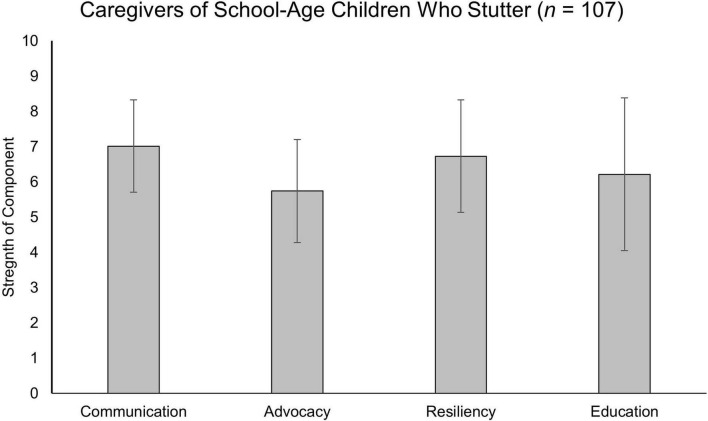
Means and +/- 1 standard deviations for the responses of caregivers of school-age children who stutter (*N =* 107) to questions relating to each of the four components of the Revised (2026) CARE Model Assessment instrument (0 = low strength and 10 = high strength).

Figure 4 illustrates the means and standard deviations for the responses of the children’s caregivers (*N* = 107) to the Revised (2026) CARE Model Assessment instrument for all four components. These responses suggest that caregivers also respond at a similar level (M range: 5.7–7.0) across the four components. Caregiver ratings of their child’s overall communication competence (I-A) were *M* = 72.83, *SD* = 16.56 (100-point VAS).

#### 4.4.2 Preliminary statistical findings

The following provides a recap of (1) our four hypotheses, (2) the possible implications of their support, and (3) the inferential statistical results testing each hypothesis.

##### 4.4.2.1 Hypothesis 1: consistency of school-age children’s self-reported responses

Our first hypothesis (H1) was that the scores of school-age children who stutter would show statistically significant correlations for all four components across two administrations of the Revised (2026) CARE Model Assessment, in the absence of any intervening treatment (*M* = 3.11 months, *SD* = 1.89 months). These four components correspond with the four subscales of the Revised (2026) CARE Model Assessment [i.e., Communication Assessment (I-C), Advocacy Assessment (II-B), Resiliency Assessment (III-B), and Education Assessment (IV-B)].

###### 4.4.2.1.1 Test-retest reliability

**FIGURE 5:**
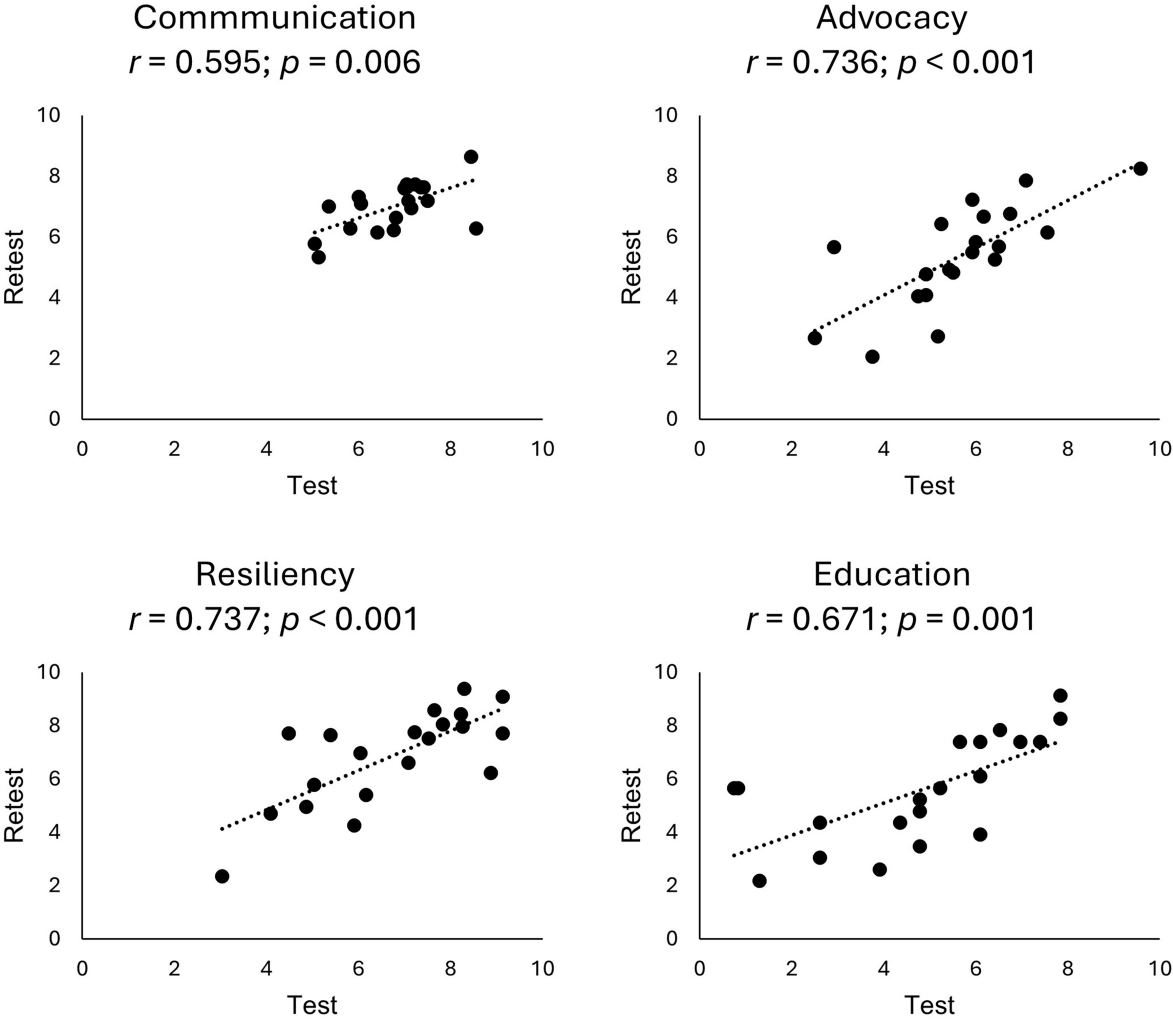
Examined for hypothesis 1 (*H1*): consistency of children’s responses during test–retest (*N =* 20) for sections I-C (*Communication Assessment*), II-B (*Advocacy Assessment*), III-B (*Resiliency Assessment*), and IV-B (*Education Assessment*).

Our analysis confirmed H1, demonstrating significant test–retest correlations among school-age children who stutter (N = 20) across the four components of the Revised (2026) CARE Model Assessment instrument: Communication (r = 0.595; p = 0.006), Advocacy (r = 0.736; p < 0.001), Resiliency (r = 0.737; p < 0.001), and Education (r = 0.671; p = 0.001) (see Figure 5). These findings suggest that children’s responses to the Revised (2026) CARE Model Assessment instrument (i.e., test–retest reliability) remain reasonably stable across administrations.

###### 4.4.2.1.2 Item analysis (Cronbach’s analysis)

We employed Cronbach’s alpha to evaluate the internal consistency of Communication, Advocacy, Resilience, and Education components. A Cronbach’s alpha >0.80 is generally considered to reflect strong internal consistency among items. All four components demonstrated strong reliability (Communication: α = 0.93; Advocacy: α = 0.80; Resiliency: α = 0.96; Education: α = 0.81), indicating internal coherence among items. We also examined item-level diagnostics using “alpha if item deleted” statistics. No single item substantially improved the overall alpha when removed from the Communication subscale (α range: 0.92 to 0.93), the Advocacy subscale (α range: 0.77 to 0.84), the Resilience subscale (α range: 0.96 to 0.96), or the Education subscale (α range: 0.79–0.82).

Overall, these findings provide empirical support for retaining all items for each of the four components of the Revised (2026) CARE Model Assessment instrument. Further, these findings confirm the instrument’s scale’s stability across all four components.

##### 4.4.2.2 Hypothesis 2: consistency of perceptual ratings of communication competence

Our second hypothesis (H2) was that school-age children’s self-ratings of their (1) overall communication competence, (2) communication competence during dyadic interactions, and (3) communication competence during oral presentations would remain significantly correlated across two administrations of the Revised (2026) CARE Model Assessment in the absence of intervening treatment. These three ratings (overall, dyadic interactions, and oral presentations) are scored on a 100-point VAS and correspond with the three subsections of the Communication components of the Revised (2026) CARE Model Assessment (i.e., I-A, Free Response & Overall Communication, and I-B Communication Activities). These three ratings are captured prior to completing section I-C (Communication Assessment), which was examined for test–retest reliability in H1.

H2 also examined clinicians’ ratings of children’s communication competence during dyadic interactions and oral presentations (subsection I-B). If the analysis supports H2, this suggests that children’s and clinicians’ ratings of communication competence across contexts, as assessed using the Revised (2026) CARE Model Assessment instrument (i.e., test–retest reliability), remain reasonably stable from one administration to another in the absence of intervening treatment sessions.

###### 4.4.2.2.1 Test-retest reliability

**FIGURE 6:**
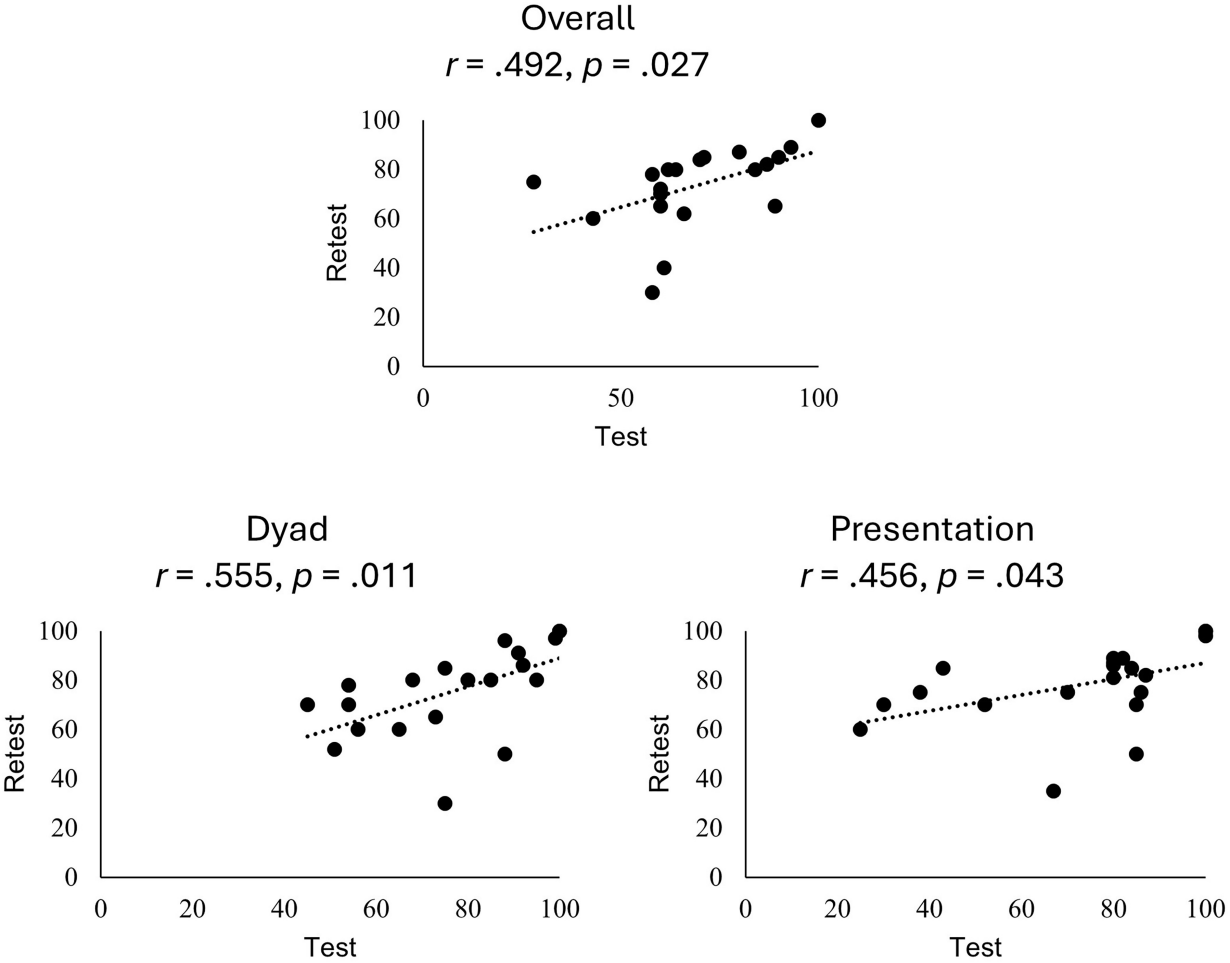
Examined for hypothesis 2 (*H2*): consistency of children’s self-ratings during test–retest (*N =* 20) for sections I-A (*Overall Communication*) and I-B (*Communication Activities*).

Our analysis confirmed H2. Overall self-ratings of communication competence were significantly correlated (r = 0.492, p = 0.027) across the two administrations of the Revised (2026) CARE Model Assessment. Similarly, children’s self-ratings of communication competence during dyadic interactions (r = 0.555, p = 0.011) and oral presentations (r = 0.456, p = 0.043) were significantly correlated across the two administrations. Clinicians’ ratings of children’s communication competence during dyadic exchanges (r = 0.48, p = 0.034) and oral presentations (r = 0.50, p = 0.028) were also significantly correlated between administrations (see Figure 6).

###### 4.4.2.2.2 Interrater reliability of clinician ratings (intra-class correlation, or ICC)

We conducted an interrater reliability analysis to assess the consistency of clinicians’ communication ratings across the dyad and presentation samples. Two clinicians, a primary coder and a second coder, independently rated all dyadic and presentation samples at each time point. The analysis found that clinician ratings of communication competence during dyadic interactions demonstrated statistically significant interrater reliability [ICC = 0.88; 95% CI (0.48, 0.97); p = 0.003], as did their ratings during oral presentations [ICC = 0.82; 95% CI (0.26, 0.95), p = 0.010]. These findings confirm the interrater reliability of clinician ratings of communication competence for dyad and presentation samples.

This analysis demonstrates that children’s self-ratings and clinicians’ ratings of communication competence across contexts, as assessed by the Revised (2026) CARE Model Assessment instrument (i.e., test–retest reliability), remain reasonably stable from one administration to another.

##### 4.4.2.3 Hypothesis 3: relation between communication competence and stuttering severity

Our third hypothesis (H3) posited that there would be no statistically significant correlation between clinicians’ judgments of the (a) communication competence and (b) severity of stuttering in school-age children who stutter. Recall that the CARE Assessment does not include ratings of stuttering severity, but this measure was employed in the present study to test the hypothesis underlying one of the CARE Model’s basic tenets, which posits that communication competence can be strengthened independent of targeting speech fluency. To let their internal criteria freely vary, raters in this study, as in previous studies (e.g., Byrd et al., 2024b; Coalson and Byrd, 2025; Coalson et al., 2024; Erickson and Block, 2013; Werle et al., 2021), were not provided with a definition of communication from which to anchor their judgment.

###### 4.4.2.3.1 Divergent validity

**FIGURE 7:**
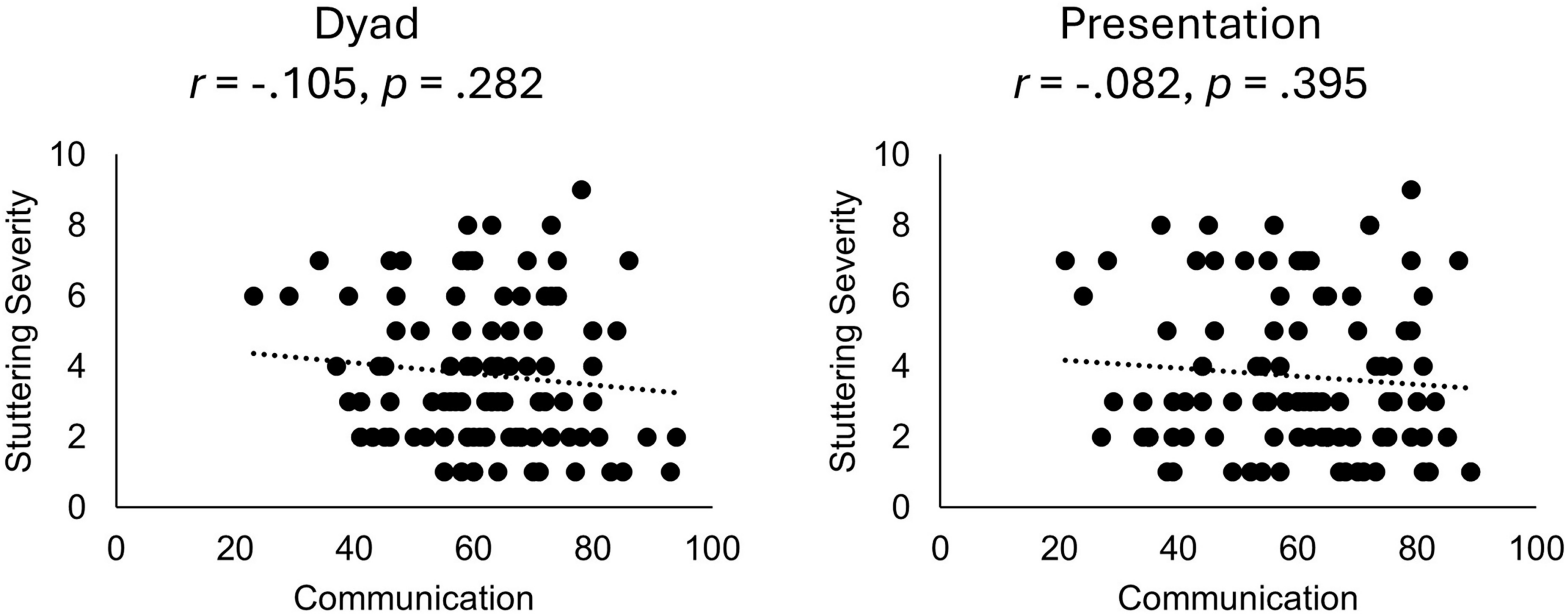
Examined for hypothesis 3 (*H3*): correlation between communication and stuttering severity ratings.

H3 was confirmed. Findings indicate no significant association between clinicians’ judgments of school-age children’s (N = 107) stuttering severity and communication competence in either speaking context (see Figure 7). Specifically, there were no significant correlations between clinicians’ ratings of school-age children’s stuttering severity and clinician’s ratings of overall communication during both dyadic interaction (r = −0.105, p = 0.282) and oral presentations (r = −0.082, p = 0.395).

###### 4.4.2.3.2 Interrater reliability of clinician ratings

We conducted an interrater reliability analysis to confirm the reliability of clinicians’ ratings of (1) communication competence and (2) stuttering severity for dyad and presentation samples. Two clinicians, a primary coder and a second coder (the same individuals as in H2), independently rated a subset of the total cohort (N = 27, or 25% of 107). The analysis demonstrated that the clinician ratings were reliable. Specifically, clinicians’ ratings of communication competence during dyadic interactions demonstrated statistically significant reliability [ICC = 0.81; 95% CI (0.58, 0.91), p < 0.001], as were their ratings of oral presentations [ICC = 0.84; 95% CI (0.64, 0.93), p < 0.001]. Furthermore, clinicians’ stuttering severity ratings for dyadic interactions [ICC = 0.91; 95% CI (0.81, 0.96), p < 0.001] and oral presentations [ICC = 0.93; 95% CI (0.84, 0.97), p < 0.001] exhibited significant interrater reliability.

Overall, these findings indicate reasonable stability of clinicians’ ratings of communication competence and stuttering severity for the Revised (2026) CARE Model Assessment instrument’s dyad and presentation items. Importantly, the findings further demonstrate that these ratings are independent, with no significant relationship between stuttering severity and communication competence.

##### 4.4.2.4 Hypothesis 4: relation between the four CARE assessment components

Our fourth hypothesis (H4) posited that, for all school-age children who stutter and their caregivers, there would be significant correlations among the four components of the Revised (2026) CARE Model Assessment instrument, without any component being functionally dependent on another (i.e., no component serves as a proxy for any other component).

###### 4.4.2.4.1 Redundancy analysis

H4 was confirmed. Findings demonstrated statistically significant correlations among all four components for school-age children who stutter (Table 2; r-values: 0.26–0.61; p-values: < 0.001–0.006). Three of the four components were significantly correlated for the children’s caregivers (r-values: 0.41, 0.46, 0.68; p-values < 0.001). Education, however, was not correlated with the ratings of the other components on the caregiver proxy (Communication: p = 0.950; Advocacy: p = 0.849; Resiliency: p = 0.099). Notably, we detected no multicollinearity among any components for either school-age children or their caregivers. Multicollinearity is indicated by variable inflation factors (VIFs). As an index of multicollinearity, a VIF reflects the degree to which information in each section overlaps with that in other sections [i.e., the four components of the Revised (2026) CARE Model Assessment], with values > 4 indicating excessive multicollinearity (Cohen et al., 2003).

**TABLE 2:** Examined for hypothesis 4 (H4): correlations and multicollinearity between components of the Revised (2026) CARE Model Assessment as reported by school-age children and their caregivers.

CARE component	Communication	Advocacy	Resiliency	Education	VIF
Children (N = 107)					
Communication	1.0				1.74
Advocacy	0.51***	1.0			1.57
Resiliency	0.61***	0.51***	1.0		1.78
Education	0.26**	0.37**	0.35**	1.0	1.21
Caregivers (N = 107)					
Communication	1.0				1.95
Advocacy	0.41***	1.0			1.30
Resiliency	0.68***	0.46***	1.0		2.14
Education	−0.01	0.02	0.16	1.0	1.05

**p <0.01, ***p < 0.001. VIF, variance inflation factor; values lower than 4.0 indicate low multicollinearity.

Findings indicate that VIF values did not exceed the threshold of 4 for either school-age children (1.21–1.78) or their caregivers (1.06–2.14) for all four components. This suggests that the components are sufficiently independent from one another during assessment. That is, despite being interrelated, none of the four components is redundant with any of the others. Thus, their collinearity is low (below 4.0) because each appears to stand on its own. Child and caregiver responses were also significantly correlated across subscales (Communication: r = 0.33, p < 0.001; Advocacy: r = 0.24, p = 0.011; Resiliency: r = 0.24, p = 0.012; Education: r = 0.40, p < 0.001).

###### 4.4.2.4.2 Ancillary analysis (H4)

We performed an exploratory analysis to investigate why the Education component was uniquely uncorrelated between caregivers and children. Specifically, we examined whether the overall scores of children and caregivers were dissimilar for Education relative to the remaining components of the Revised (2026) CARE Model Assessment. We conducted a series of four paired-sample t-tests, applying Bonferroni-adjusted p-values to account for multiple comparisons (0.05/4 = 0.0125). As summarized in Table 3, caregivers scored significantly higher than their children on the Education subscale (p = 0.004). This suggests that caregivers’ overall knowledge of stuttering was more accurate than that of their school-age children. In the discussion section, we provide insight into this small yet significant difference in caregiver scores.

**TABLE 3:** Child (*N* = 107) and caregiver (*N* = 107) self-ratings for the Revised (2026) CARE Model Assessment.

CARE component	Children	Caregiver	*t*	*p*	*d*
Communication	6.71 (0.15)	7.01 (0.13)	−1.48	0.140	
Advocacy	5.77 (0.17)	5.74 (0.14)	0.121	0.904	
Resiliency	6.18 (0.23)	6.72 (0.15)	−2.03	0.043^†^	
Education	5.35 (0.21)	6.21 (0.21)	−2.92	0.004	0.40 (small)

Bonferroni-adjusted *p*-value applied to account for multiple comparisons. Values reflect sample means. Standard error of mean in parentheses.

† Did not exceed Bonferroni-corrected *p*-value (0.05/4 = 0.0125).

Overall, these findings demonstrate associations among the clinical outcomes within each of the four components of the Revised (2026) CARE Model Assessment, as expected, but remain sufficiently independent.

## 5 Discussion

### 5.1 Studies 1 and 2—general implications

#### 5.1.1 Study 1

The findings of Study 1—the development of the Revised (2026) CARE Model Assessment instrument—suggest that the instrument is methodologically and statistically sound for assessing the four components of the CARE Model. This conclusion is based on extensive community-based participatory research: (1) Community stakeholders, both familiar and unfamiliar with the CARE Model, (2) a subsample of the same individuals who were administered both the initial and revised versions of the CARE Assessment instrument, and (3) individuals who had not previously completed the initial version.

As previously discussed, following the results of administering the Initial (2023) CARE Model Assessment instrument, we made a few additional minor wording changes. We also incorporated culturally responsive instructions for the examiner of the Revised (2026) CARE Model Assessment instrument to facilitate their adaptation of the questionnaire items, as needed on a case-by-case basis. For example, instructing the examiner not to evaluate eye contact for those for whom it may not be neurodevelopmentally or culturally applicable. This draft was then re-reviewed by all stakeholders (i.e., teachers, clinicians, caregivers, children, teens, and adults who stutter) and re-administered by the same familiar and unfamiliar SLP cohorts to the same subsample of participants.

The iterative administrations of the Revised (2026) CARE Model Assessment instrument in Study 1 led to its refinement and consolidation regarding (1) language and phrasing, (2) definition of terms, (3) reduction in redundancy, (4) reduction in length, and (5) context and scale for assessing communication, as well as for other variables germane to the instrument and assessment. Study 1’s findings served as the basis for the experimental testing of the four primary hypotheses in Study 2.

#### 5.1.2 Study 2

Study 2’s results— Responses to the Revised (2026) CARE Model Assessment instrument regarding all four CARE components [i.e., Communication (I-C), Advocacy (II-B), Resiliency (III-B), and Education (IV-B)]—support the following four statements regarding school-age children who stutter. These children (1) are consistent in their test and retest responses, (2) are consistent in their test and retest self-ratings of overall as well as situational communication competence, as are their clinicians’ ratings, (3) exhibit neither a descriptive nor a statistically determined relationship between communication and stuttering severity and (4) demonstrate significant correlations among the four CARE Assessment components. The results also showed that there was no significant redundancy in response variability across the four components.

Overall, the findings from Study 2 suggest that the Revised (2026) CARE Model Assessment instrument demonstrates good test–retest reliability from the perspectives of both school-age children who stutter and clinicians. Additionally, the instrument’s Communication competence component was unrelated to stuttering severity, and all four components of the instrument (Communication, Advocacy, Resiliency, and Education) were interrelated but not so closely associated with one another that any component lacked independent value. More detailed discussions of each of these findings are provided below.

### 5.2 Study 2’s four hypotheses—specific implications

#### 5.2.1 Test–retest reliability of self-ratings (H1)

Typically, days or months elapse between the initial assessment and the start of treatment. When such delays occur, clinicians may wonder if a school-age child’s initial assessment scores remain relevant. The present findings provide a preliminary degree of confidence that different administrations of the Revised (2026) CARE Model Assessment, when delivered at two separate time points (*M* = 3 months), are fairly comparable in the absence of any intervening treatment. Note that the test-retest sample size is small (*N* = 20), which limits generalizability. Nevertheless, the findings indicate that clinicians can be confident that school-age children’s responses to the Revised (2026) CARE Model Assessment instrument will remain stable between completion of the assessment and initiation of treatment for a reasonable period of time (i.e., 3 months).

#### 5.2.2 Test-retest reliability of communication competence (H2)

Clinicians may also wonder whether communication competence scores, particularly the self-ratings of children who stutter and the observations of clinicians, remain consistent. The present results suggest that such communication competence scores are comparable and stable over time. Specifically, the responses for dyadic interactions and oral presentations, provided by both children (via self-ratings) and clinicians (via independent ratings), retain measurable reliability across the Communication component of the Revised (2026) CARE Model Assessment (i.e., Communication Activities, I-B). Furthermore, children’s self-ratings of their overall communication (i.e., Overall Communication, I-A) were consistent between test–retest administrations. An empirical study with a larger sample and additional replications is warranted; however, the test–retest data from the present, preliminary cohort are promising. Findings pertaining to H2 suggest that, similar to child self-ratings, clinicians may regard ratings of communication competence as sufficiently stable.

#### 5.2.3 Correlation between communication and stuttering severity (H3)

Central to the rationale for the CARE Model is that strengthening communication does not necessitate increased fluency. We previously reported empirical evidence supporting the claim that communication and stuttering are independent of one another (Byrd et al., 2024b; Coalson et al., 2024; Coalson and Byrd, 2025; Werle et al., 2021). Indeed, our prior, statistically supported findings are consistent with our present empirical findings of no apparent relationship between ratings of communication competence and that of severity.

#### 5.2.4 Relation between the four CARE assessment components (H4)

The present findings confirm that all four components of the Revised (2026) CARE Model Assessment instrument are related to one another but are not collinear. This suggests that no single component can fully predict another—while communication, advocacy, resiliency, and education are statistically associated with one another (based on the self-ratings of school-age children who stutter), each component stands on its own, providing non-redundant, task-specific insight for assessment.

From the caregivers’ perspective, these components are similarly interrelated, except for Education (see Table 2). Unlike their children, caregivers’ Education component scores were not related to their perceptions of their child’s Communication, Advocacy, or Resiliency. Table 3 displays the results of analyses comparing child and caregiver self-ratings across the four components. These findings suggest that caregivers were significantly more accurate on the Education component than their children. We believe that these findings may suggest that caregivers’ accurate knowledge of stuttering does not drive their perceptions of their child’s ability to communicate, advocate, or exhibit resilience. Conversely, children’s less accurate knowledge about stuttering may influence their self-perceived communication and advocacy skills to a greater degree.

The lack of correlation between Education and the other components, as well as the low collinearity, further indicates that the Education measures do not overlap, unlike the other three subscales. The Education component of the Revised (2026) CARE Model Assessment instrument (which assesses knowledge about stuttering) is measured based on caregiver responses. Unlike the other sections of the assessment, which ask caregivers to share what they think their child would share, the Education section asks caregivers about their own knowledge rather than asking them to take their child’s perspective of their child’s knowledge. Thus, the lack of correlation for the Education component of the Revised (2026) CARE Model Assessment instrument makes sense, at least prior to the intervention. Future studies comparing pre- and post-treatment should explore increased knowledge and alignment between the caregiver and the child.

### 5.3 Summary

While preliminary, the present findings yield a straightforward interpretation. First, they show that school-age children’s responses to the Revised (2026) CARE Model Assessment instrument demonstrate reasonable stability across test–retest administrations. Second, the stuttering severity of school-age children is not significantly associated with their responses to the Communication competence component of the assessment. Finally, except for the Education component, the other three components of the Revised (2026) CARE Model Assessment appear to be interrelated. Critically, however, no single component is redundant with another; despite their associations, they are not significantly predictive of one another. The descriptive and inferential statistical evidence presented suggests that the Revised (2026) CARE Model Assessment instrument provides predictable, stable, and useful information for those who may subsequently use it develop a strengths-based treatment plan for school-aged children.

### 5.4 Additional considerations and future studies

As with all empirical studies, these findings come with caveats. First, they are preliminary and therefore indicative rather than conclusive in their implications. Future studies, employing larger sample sizes, findings from related studies, and further considering the demographics of school-age children and their caregivers, may provide additional nuance to the present results. Additional studies examining administration of this instrument are warranted to assess potential variance in responses across different clinical settings and cultures.

Second, this study focused on the inception, development, refinement, and initial findings of the Revised (2026) CARE Model Assessment instrument independent of implementing any stuttering-affirming treatments. Therefore, the study does not include pre-treatment versus post-treatment comparisons. Future studies will undertake such comparative analyses as a separate study, one we are currently conducting with a larger sample of school-age children and their caregivers.

Third, the present study reports ratings only for the closed-ended questions specific to each component. It does not include the data from the open-ended questions that form part of the Revised (2026) CARE Model Assessment instrument. We are currently analyzing open-ended responses from the 107 participants and their caregivers to identify distinct themes relevant to each of the four components and determine the stability of the responses from test to retest, as well as their association with quantitative scores. Additional detailed analyses of communication competencies ratings provided by children and clinicians, along with responses from the teacher proxy questionnaires, will be conducted in future studies.

## 6 Conclusion

This study provided preliminary empirical evidence on the initiation and implementation of the Revised CARE Model Assessment instrument for school-aged children. This instrument enables a thorough assessment of an individual’s unique strengths across communication, advocacy, resiliency and education, providing additional distinct insight beyond existing instruments. The Revised CARE Assessment instrument for school-age children who stutter is the product of a sustained community-based participatory research that is person-centered. It can be utilized to directly map those individual-level insights onto strength-based interventions that are consistent with the CARE Model for treating school-age children who stutter, which ultimately aims to improve their quality of life. To conclude, we suggest that the development and preliminary evaluation of the Revised CARE Assessment instrument represent a constructive step toward planning the treatment of stuttering through a strengths-based lens.

## Author’s note

The first author (Byrd) is the Founding and Executive Director of the Arthur M. Blank Center for Stuttering Education and Research at The University of Texas at Austin, a full professor, and a certified speech-language pathologist with more than 30 years of clinical and research experience. The second author (Coalson) stutters openly, is the Associate Director of Grant and Research Development at the Arthur M. Blank Center for Stuttering Education and Research at The University of Texas at Austin, a parent of someone who stutters openly, and a certified speech-language pathologist with over 15 years of clinical and research experience. The third author (Conture) is the Senior Director of Grant and Research Development at the Arthur M. Blank Center for Stuttering Education and Research at The University of Texas at Austin, a retired speech-language pathologist, and a Professor Emeritus from Vanderbilt University with more than 50 years of clinical and research experience. All the authors have experience developing and/or administering CARE Model Assessment and Treatment with children and adults, which motivates their combined interest in investigating its clinical efficacy.

## Data Availability

The datasets presented in this study can be found in online repositories. The names of the repository/repositories and accession number(s) can be found at: https://doi.org/10.18738/T8/WSCNDS.

## Footnotes

1 Demographic information for race and ethnicity was based on classifications adopted by the National Institutes of Health in 2015 (National Institutes of Health [NIH], 2015), which categorize individuals according to five racial groups (i.e., American Indian or Alaskan Native, Asian, Black or African American, Native Hawaiian or Other Pacific Islander, and White) and using a separate, binary ethnic classification (i.e., Hispanic or Latino, Not Hispanic or Latino). We acknowledge that these categories do not adequately reflect the full spectrum of racial and/or ethnic identity. Updated protocol and categories for describing race and ethnicity as a combined, single question were adopted as USA federal standards for data collection in March 2024 (U.S. Office of Management Budget [U.S. OMB], 2024; i.e., American Indian or Alaskan Native, Asian, Black or African American, Native Hawaiian or Other Pacific Islander, and White). Although the Arthur M. Blank Center has since adopted these newer categories, we continue to acknowledge that these categories, and the examples or descriptions provided, do not fully capture racial and/or ethnic identity.
